# Wrinkles, Ridges, Miura-Ori, and Moiré Patterns in MoSe_2_ Using Neural Networks

**DOI:** 10.1021/acs.jpclett.2c03539

**Published:** 2023-02-09

**Authors:** Anikeya Aditya, Ankit Mishra, Nitish Baradwaj, Ken-ichi Nomura, Aiichiro Nakano, Priya Vashishta, Rajiv K. Kalia

**Affiliations:** Collaboratory for Advanced Computing and Simulations, Department of Chemical Engineering and Materials Science, Department of Physics & Astronomy, and Department of Computer Science, University of Southern California, Los Angeles, California 90089, United States

## Abstract

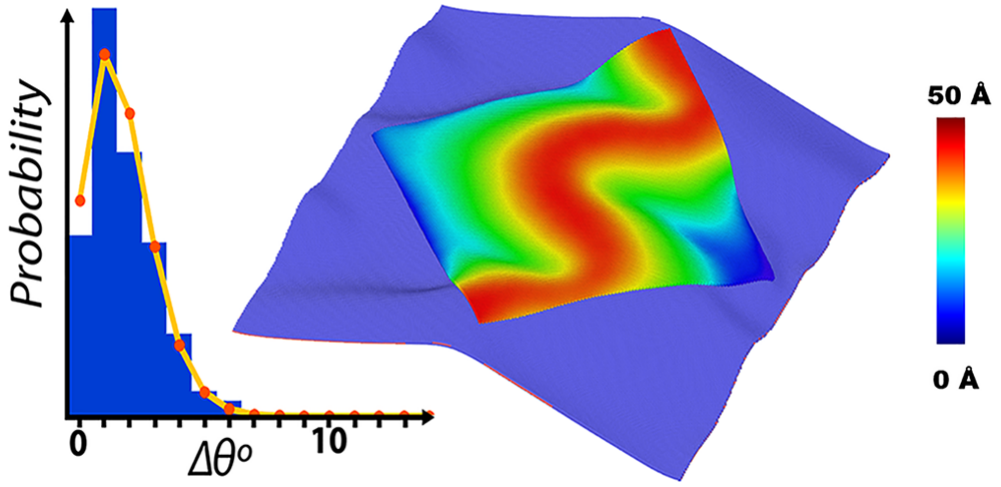

Effects of lateral compression on out-of-plane deformation of two-dimensional MoSe_2_ layers are investigated. A MoSe_2_ monolayer develops periodic wrinkles under uniaxial compression and Miura-Ori patterns under biaxial compression. When a flat MoSe_2_ monolayer is placed on top of a wrinkled MoSe_2_ layer, the van der Waals (vdW) interaction transforms wrinkles into ridges and generates mixed 2H and 1T phases and chain-like defects. Under a biaxial strain, the vdW interaction induces regions of Miura-Ori patterns in bilayers. Strained systems analyzed using a convolutional neural network show that the compressed system consists of semiconducting 2H and metallic 1T phases. The energetics, mechanical response, defect structure, and dynamics are analyzed as bilayers undergo wrinkle–ridge transformations under uniaxial compression and moiré transformations under biaxial compression. Our results indicate that in-plane compression can induce self-assembly of out-of-plane metasurfaces with controllable semiconducting and metallic phases and moiré patterns with unique optoelectronic properties.

Graphene and two-dimensional (2D) transition metal dichalcogenides (TMDCs) display exceptional robustness and flexibility due to high in-plane and low out-of-plane stiffness.^1^ The flexibility of 2D materials can be quantified by the Föppl–von Kármán number, which is proportional to the ratio of the in-plane Young’s modulus to bending stiffness.^2^ This number is extremely large for graphene and 2D TDMCs, and hence, they are as flexible as a sheet of paper.

The flexibility of 2D materials has been exploited to design kirigami and origami structures. Cai et al. have used chemical vapor deposition followed by chemical etching to design exotic kirigami structures of 2D WSe_2_.^3^ Experiments, modeling and simulation, and machine learning have been used to design kirigami structures of graphene and 2D MoS_2_ that are highly stretchable and recoverable upon loading and unloading.^2,4^ Two-dimensional materials can also be folded quite easily. Zhao et al. demonstrated this experimentally using directed fluid flow.^5^ They could control the folding geometry, direction, and position of 2D materials placed on polymer substrates in a microfluidic environment, and they were able to determine the underlying mechanism and energetics of folding using molecular dynamics (MD) simulations. Their joint experimental–simulation study has paved the way for the design of vertically stacked van der Waals (vdW) architectures in a microfluidic environment. These architectures exhibit a wide range of exotic phenomena such as superconductivity, atomic photonic crystals, topological excitons in stacked TMDCs, magnetism, and Mott transition associated with moiré physics.^6−18^

In this paper, we examine if ultrathin MoSe_2_ sheets can form complex structures like wrinkles, ridges, and Miura-Ori patterns akin to those seen in nature during the opening and closing of leaves and wings of insects.^19−21^ Wrinkles were first observed in a thin metallic film on a polydimethylsiloxane substrate when the system was heated and then cooled to ambient temperature.^22^ It was argued that wrinkle formation was caused by compressive stress redistribution due to the buckling of stiff outer films, and it was shown that the dimensions of wrinkles could be controlled by the substrate structure.^21^ In subsequent experimental and simulation studies, period doubling of wrinkles and wrinkle-to-ridge transformation were seen in compressively strained thin films supported on soft substrates.^19,21,23−28^

We have performed MD simulations to examine the effects of lateral compression on monolayer and bilayer MoSe_2_.^29−32^ We use a convolutional neural network (CNN) trained on a prior MD data set of MoWSe_2_ to demonstrate the viability of transfer learning in analyzing structural changes in strained monolayer and bilayer MoSe_2_.^33,34^ We find that an unstrained MoSe_2_ monolayer in the semiconducting 2H phase develops wrinkles under uniaxial compression and Miura-Ori patterns under biaxial compression. The wrinkled sheets are hybrid structures consisting of the 2H phase in low-stress regions around the peaks and valleys and the 1T phase in the high-stress slopy regions. Next, we study the effect of wrinkles and Miura folds on pristine overlayers of MoSe_2_. When we place flat MoSe_2_ monolayers above wrinkled MoSe_2_ monolayers, the bilayers bind by vdW interaction and wrinkles transform into ridges. The wrinkle–ridge transformation is accompanied by the appearance of mixed 2H and 1T phases and chain-like defects of Mo and Se atoms. In the case of biaxial strain, the vdW interaction induces a commensurate Miura-Ori pattern in the overlayer. The Miura-Ori patterns form novel moiré patterns under relative twisting of the two monolayers. The energetics, thermomechanical behavior, and defect dynamics are analyzed in MoSe_2_ bilayers as they undergo wrinkle–ridge transformations in uniaxially strained systems and moiré transformations of Miura folds in biaxially strained bilayers.

We have investigated the effect of vdW interaction on the formation of wrinkles, ridges, Miura folds, and moiré patterns in MoSe_2_ bilayers. We simulated many bilayers with dimensions ranging from 400 Å × 200 Å to 3200 Å × 1600 Å. Here we present results for the largest system. Additional results are presented in the Supporting Information. We provide a video in the Supporting Information that shows the formation of wrinkles and Miura-Ori patterns and the emergence of 2H and 1T patterns. Figure 1 shows structural changes in MoSe_2_ monolayers subjected to in-plane uniaxial and biaxial strains. In both cases, the compressive strain is 6% and the temperature is 300 K. Figure 1a shows out-of-plane atomic displacements in a uniaxially strained MoSe_2_ monolayer. The displacements appear in the form of periodic wrinkles in the direction of compression (*x*). Blue regions represent valleys below the unstrained monolayer, and red regions indicate wrinkle peaks. The wrinkle amplitude is ∼130 Å. The wrinkles result from inhomogeneous stress distributions, which are shown in Figure S3. Peaks and valleys have smaller stresses than the slopes of wrinkles. The CNN analysis of atomic configurations is presented in panels c and d of Figure 1. Under uniaxial compression, the monolayer becomes a hybrid structure of semiconducting (2H) and metallic (1T) phases that are colored blue and cyan, respectively. In Figure 1c, the 2H phase exists in small stress regions, i.e., peaks and valleys of wrinkles in which the top and bottom Se layers have shifted slightly relative to each other. Larger tensile stresses on the slopes of wrinkles induce a 2H → 1T phase transformation by changing the Se–Mo–Se stacking from ABA to ABC. At higher strains, we observe period doubling of wrinkles that has also been seen experimentally in thin films supported on soft substrates.^19^

**Figure 1:**
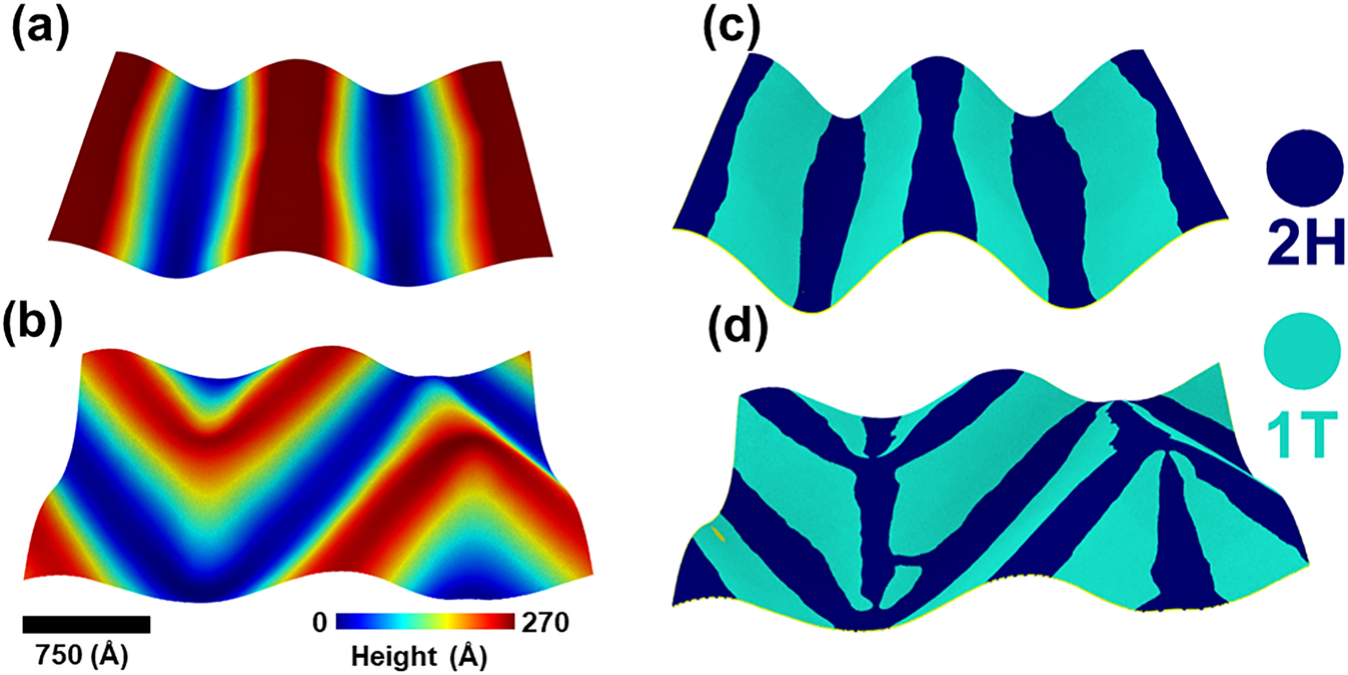
Effects of uniaxial and biaxial strains on a MoSe_2_ monolayer. Here the applied strain is 6%, and the systems are at room temperature. Panels a and b show out-of-plane displacements in uniaxially and biaxially compressed systems, respectively. Peaks and valleys are colored red and blue, respectively. Periodic wrinkles are formed under uniaxial strain, and Miura-Ori patterns under biaxial strains. Inhomogeneous stress distributions induce structural transformations in strained monolayers. Stresses are small in magnitude in valleys and peaks, and hence, the structure remains in the 2H phase, although it is slightly strained (see panels c and d). Large stresses on the slopes of wrinkles and Miura folds induce 2H to 1T phase transformation under uniaxial and biaxial strains.

Figure 1b shows that biaxial compression generates Miura-Ori patterns in the monolayer. The corresponding inhomogeneous stresses in the monolayer are shown in Figure S3. These stresses give rise to interesting patterns of 2H and 1T phases. The CNN analysis reveals the presence of the 1T phase along with point and line defects in high-stress regions. The line defects are chains of 5- and 7-fold coordinated Mo atoms and 4-fold coordinated Se atoms. These defects, called α and β, were first seen in scanning transmission electron microscopy (STEM) images of MoS_2_.^35^

The vdW interaction induces significant structural changes in MoSe_2_ bilayers. Wrinkles in a uniaxially strained monolayer disappear, and a ridge appears when the strained and pristine layers bind (see Figure S4). The ridges run straight across the bilayer in uniaxially compressed MoSe_2_. Figure S5 shows that the ridge height increases with an increase in strain because atoms tend to move from high-stress to low-stress regions, i.e., to the top of ridges. Figure S6 shows that an increase in temperature decreases the ridge height because of an increase in stress. The widths of ridges increase with increases in strain and temperature.

Vertically stacked vdW multilayers show interesting effects from compressive and uniaxial stretching.^36^Figure 2 shows the results of the vdW interaction between a pristine MoSe_2_ monolayer placed beneath another monolayer that is under a biaxial compressive strain of 6%. The strained top monolayer has Miura folds like those in Figure 1b. The inhomogeneous stresses in the strained top layer induce a Miura-Ori pattern in the pristine layer, as well. Figure 2a shows out-of-plane deformation, *z*, in the bilayer at 200 K. The deformations range from −30 Å (dark blue) to 130 Å (brown). Large deformations in red-brown regions are separated by valleys (blue), and they occur along narrow wavy lines (green) in which the displacements along *z* are small. Figure 2b shows atomic stresses on Mo atoms in the bilayer at 200 K. The stresses are small at the peaks and valleys of out-of-plane deformations, but they are much larger along the green wavy lines of Figure 2a. The CNN analysis reveals the presence of the 2H (dark blue) and 1T (light blue) phases and defects (green) (see Figure 2c). The 2H phase is in low-stress regions, and the 1T phase is in higher-stress regions. Line defects α and β as well as point defects are found in regions of high compressive stress. Panels d–f of Figure 2 show that the system is predominantly in the 2H phase with small fractions of the 1T phase and defects. The fractions of 2H and 1T phases decrease almost linearly above 150 K, while defects increase linearly with an increase in temperature.

**Figure 2:**
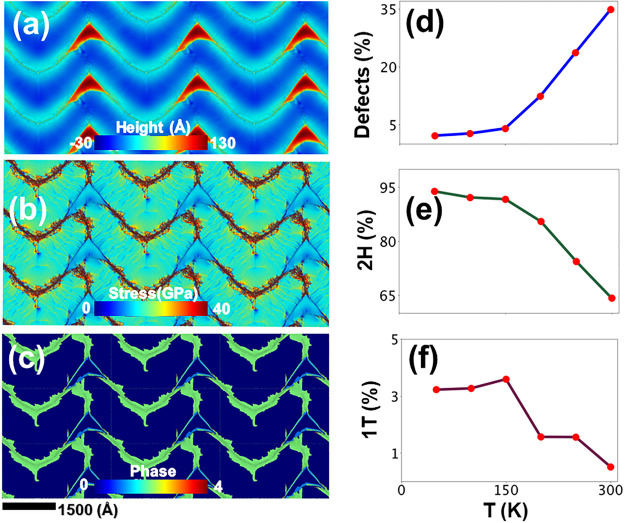
Effect of a biaxial strain (6%) on a MoSe_2_ bilayer at room temperature. Out-of-plane displacements in panel a indicate the formation of a Miura-Ori pattern in the bilayer. Panel b shows inhomogeneous stresses in Miura folds, and panel c shows that the bilayer regions where the atomic-level stresses are relatively small remain in the 2H phase. There are small regions of high stress where the bilayer undergoes 2H to 1T phase transformation. Panel c also indicates point and line defects in high-stress regions (colored yellow and red in panel b). Panel d shows a linear increase in the number of defects, and panels e and f show linear decreases in the fractions of 2H and 1T phases with an increase in temperature above 150 K.

We have examined the underlying potential energy surfaces (PES) of strained bilayers at various temperatures between 50 and 300 K. The PES were obtained by subjecting atomic configurations to steepest descent quench (SDQ) at regular time intervals of the MD trajectory. A SDQ brings the bilayers to the closest underlying potential energy minimum by removing the kinetic energy. Figure 3 shows the PES for a bilayer under biaxial compression, and Figure S6 shows the results under 6% uniaxial compression. Panels a and b of Figure 3 display Miura-Ori patterns at 200 and 300 K, respectively. They do not seem to change very much except that the valleys (blue regions) are slightly shallower at higher temperatures. However, the underlying PES reveals significant differences between the atomic configurations at 200 and 300 K. The PES plots are obtained by generating pixels on an *x*–*y* grid of the bilayer and averaging potential energies of atoms in pixels. The potential energies of pixels are plotted along the *z* axis. The dark brown regions in the Miura-Ori pattern shown in panels a and b of Figure 3 have higher potential energies, while the ridges adjacent to it have lower potential energies. The PES show deep drops in the blue wavy patterns corresponding to the valleys of Miura-Ori patterns. The decrease in potential energy is larger at higher temperatures.

**Figure 3:**
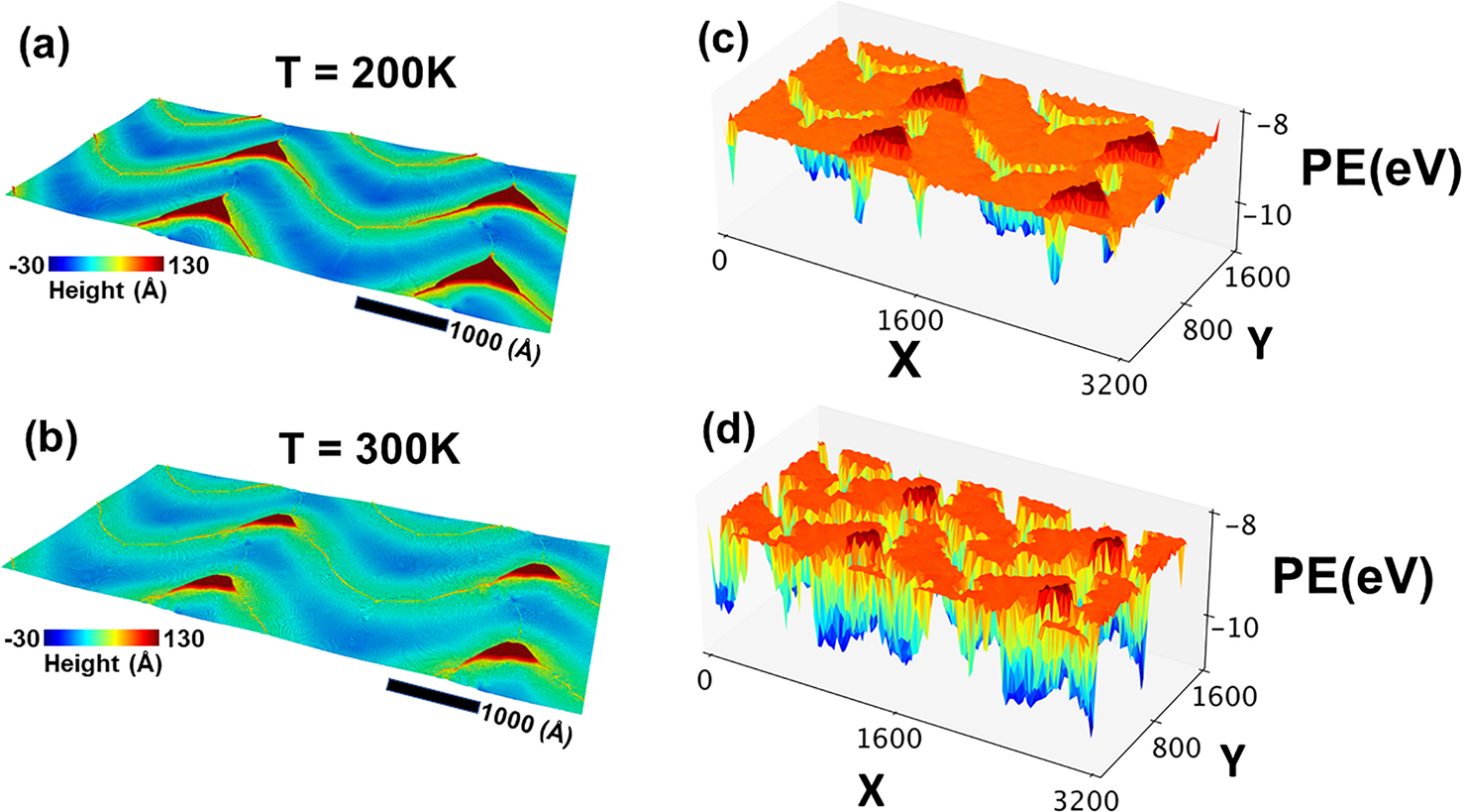
Potential energy surfaces (PES) underlying Miura-Ori patterns in a bilayer under a 6% biaxial compressive strain. Panels a and b show of out-of-plane displacements in the bilayer at temperatures of 200 and 300 K, respectively. The corresponding PES, which are the potential energies per particle averaged over all of the atoms in a pixel, are shown in panels c and d, respectively. In contrast to the out-of-plane atomic displacements, the PES show significant changes with an increase in temperature. The potential energy per pixel is high in regions of greater deformation.

In flat regions of bilayers, the two MoSe_2_ sheets are not in complete registry. They contain tiny wrinkles and interesting moiré patterns that arise from a small relative rotation between Se layers. The moiré patterns are different in the bilayers because the relative rotations of Se layers are different under uniaxial and biaxial compressions. The rotation is <2° for bilayers subjected to uniaxial compression and slightly larger for bilayers under biaxial compression. We also find tiny wrinkles in flat portions of bilayers. Figure 4a shows a small flat region of the top monolayer that was subjected to 6% biaxial compression. The close-up in Figure 4c reveals tiny wrinkles with heights of <5 Å. A tiny wrinkle also appears in the bottom layer (see panels b and d of Figure 4). The twist angle distributions shown in panels e and f of Figure 4 are similar for wrinkles in the top and bottom layers. Both are Poisson distributions with peaks around 2°. Similar types of miniscule wrinkles were seen in graphene bilayers using a superconducting quantum interference device.^37^

**Figure 4:**
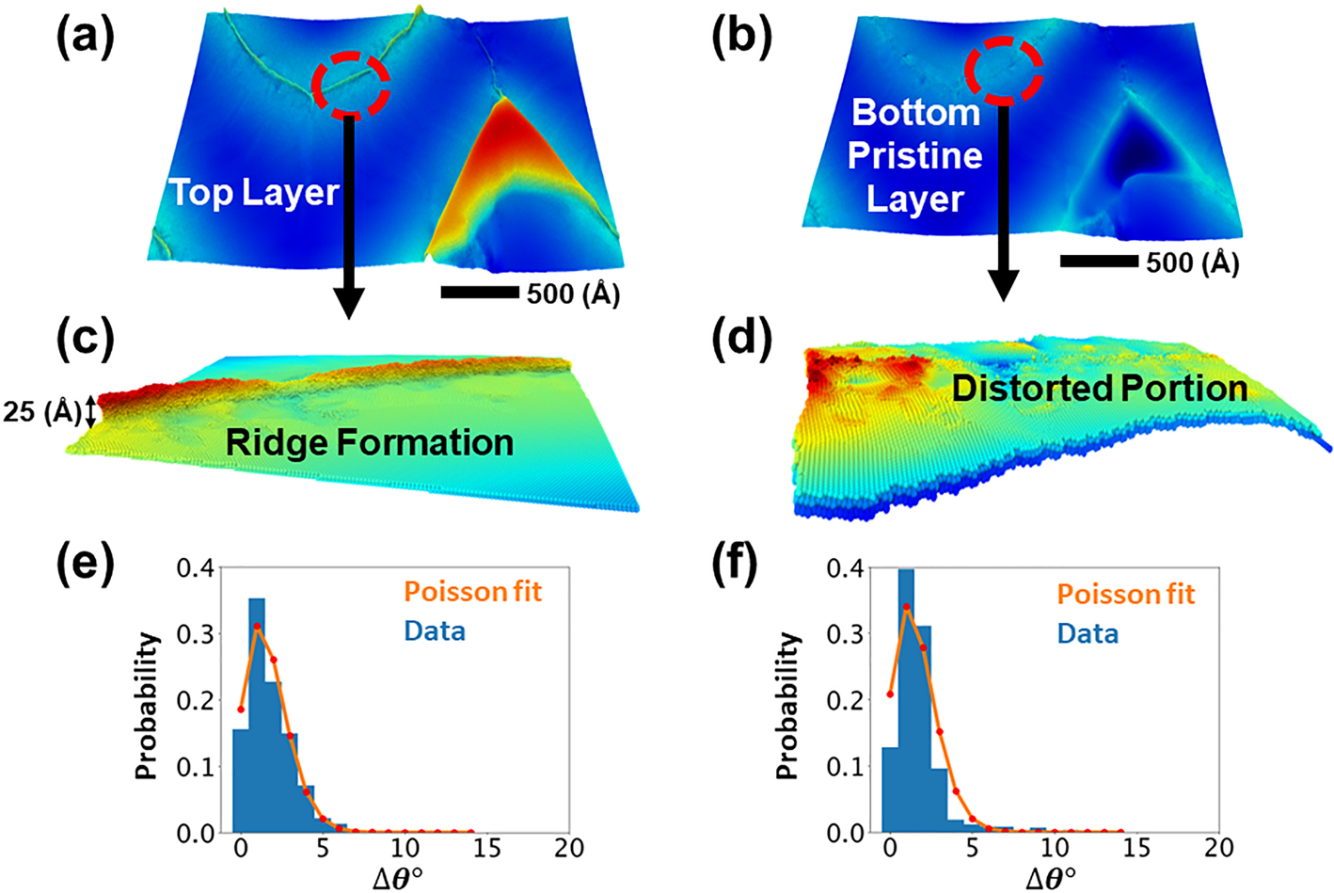
Effect of vdW interaction on Miura folds in individual monolayers of a bilayer system under a 6% compressive strain in both *x* and *y* directions. Panels a and b show a small region of a Miura fold in the bottom and top monolayers. Close-ups of that region in panels c and d, respectively, reveal a tiny wrinkle in the bottom layer that distorts the region right above it in the top monolayer. Selenium layers in the tiny wrinkle and the distorted region have twisted relative to each other. The twist angles are narrowly distributed around 2°. The Poisson distributions for the two layers are given in panels e and f, respectively.

We have examined moiré patterns in strained MoSe_2_ bilayers subjected to relative twist between the monolayers. The top MoSe_2_ monolayer was rotated relative to the bottom monolayer, and then the bilayers were relaxed by the conjugate minimization method and thermalized at different temperatures. Here we present the results for a biaxially strained system at 200 K. The system size is 800 Å × 400 Å, and the applied strain is 1%. Figure 5a shows a Miura-Ori pattern in one of the MoSe_2_ monolayers, and the close-up indicates the region in which we have examined the changes in moiré patterns with the angle of rotation. The peaks and valleys in the pattern range from −3 to 3 Å. The CNN analysis reveals a strained 2H structure in the Miura folds. Figure 5b shows the moiré pattern before twisting, and panels c–e of Figure 5 show how twisting changes those patterns. Upon twisting, larger moiré patterns appear periodically followed by smaller ones. Figure 5c shows large hexagons in the moiré pattern at a twist angle of 10°. The size of hexagons is very sensitive to the twist angle. A further increase in the twist angle first decreases and then increases the size of hexagons in moiré patterns. Panels d and e of Figure 5 show restoration of large moiré patterns at twist angles of 25° and 45°, respectively.

**Figure 5:**
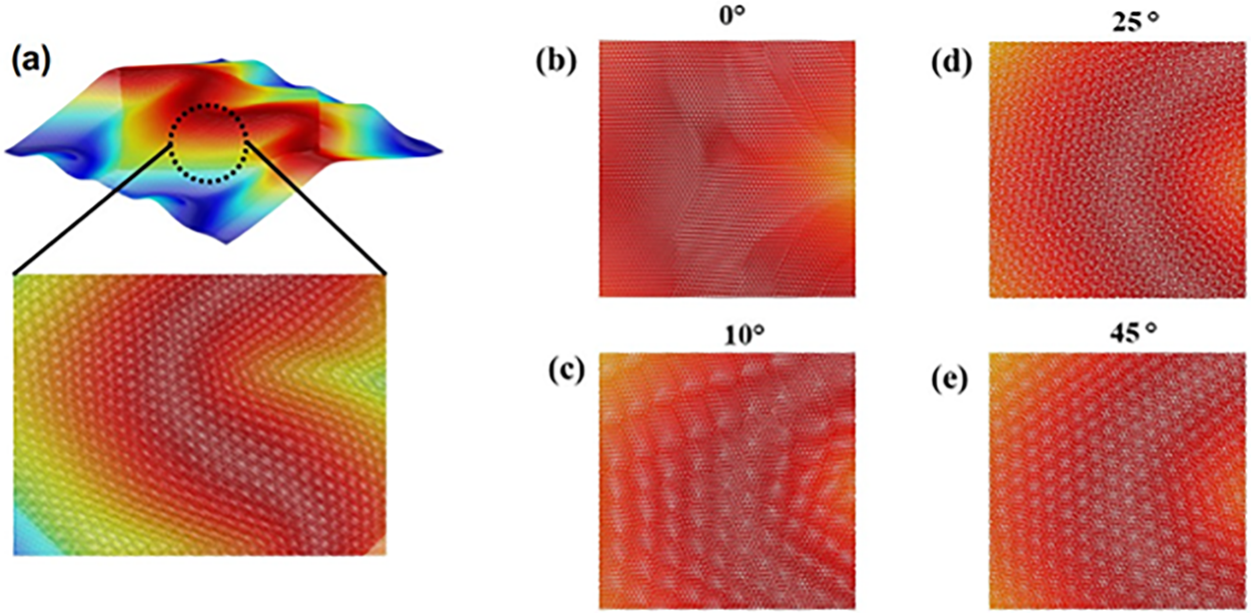
Changes in moiré patterns with the twist angle in a bilayer under a biaxial strain of 6%. Panel a indicates a close-up of a Miura fold we have examined as the top monolayer is rotated relative to the bottom layer. (b) At 0°, there is no moiré pattern. Formation of a hexagonal supercell is observed with an increase in twist angle rotation. Panel c shows hexagonal cells at 10°. (d) The size of these cells decreases upon further rotation. (e) Beyond 30° the hexagonal cells increase in size again.

In summary, 2D MoSe_2_ displays interesting structural transformations under compressive strains. In monolayers, periodic wrinkles are formed under uniaxial strain and Miura-Ori patterns under biaxial strain. When a pristine MoSe_2_ layer is vertically stacked on a uniaxially strained monolayer, the vdW interaction induces wrinkle–ridge transformation in the resulting bilayer. Biaxial strain induces commensurate and incommensurate Miura-Ori patterns in MoSe_2_ bilayers. Wrinkles, ridges, and Miura folds consist of a strained semiconducting 2H phase in which the atomic layers of Se are slightly twisted relative to each other. Twist angles are randomly distributed with peaks around 2°. In addition to the predominant 2H phase, there are small islands of the metallic 1T phase and chain-like defects in high-stress regions of wrinkles, ridges, and Miura folds. Relative rotation between the monolayers produces interesting changes in moiré patterns that are periodic functions of the twist angle.

Wrinkles, ridges, and Miura-Ori patterns can be tuned by uniaxial and biaxial strains, and in turn, they can dramatically change the electronic and optoelectronic properties of 2D materials.^38−41^ Defects present in TMDCs also impact their electronic properties.^42^ Vertical stacking and the varying composition of TMDCs with other 2D materials like graphene and hBN can tune the bandgap and exciton binding energy of these materials.^43,44^ Castellanos-Gomez et al. have examined the effect of wrinkles on optoelectronic properties through local strain engineering of atomically thin MoS_2_ supported on elastomeric substrates.^45^ They observe a red shift in the photoluminescence signal from a wrinkled MoS_2_ sheet relative to the signal from a flat monolayer, which indicates a reduction in the bandgap due to strains in the wrinkled sheet. Furthermore, they observe excitons drifting from high-stress slopes to low-stress regions on the wrinkle peaks. Through strain engineering of 2D TMDCs, it is possible to generate interesting hybrid metasurfaces consisting of metallic 1T and semiconducting 2H phases with variable bandgaps. Vertically stacked TMDCs with hybrid metallic and semiconducting metasurfaces offer exciting possibilities for nanophotonics applications.

## Computational Methods

The force field for the simulations was developed with a data set generated by density functional theory simulations, and the parameters in two- and three-body interactions were fitted to structural and mechanical properties and vibrational densities of states of unstrained and strained MoSe_2_.^46,47^ The force field was validated against quantum dynamics simulation and experimental data. Details of the force field development and validation are provided in the Supporting Information.

After validation, we performed MD simulations on unstrained MoSe_2_ monolayers with dimensions of 400 Å × 200 Å, 800 Å × 400 Å, 1000 Å × 1000 Å, 1600 Å × 800 Å, and 3200 Å × 1600 Å. These systems were relaxed using the conjugate gradient minimization scheme and then strained incrementally. Uniaxial compression transforms flat sheets into wrinkled sheets, and biaxial compression generates Miura-Ori patterns in monolayers (see Figure S3). The unstrained and strained MoSe_2_ sheets were heated to various temperatures and thermalized for 1 ns in the *NVT* ensemble, and the properties and processes in equilibrated systems were examined over 0.5 ns.

Bilayer systems were created by placing strained layers over pristine MoSe_2_ monolayers and then relaxed with the conjugate gradient minimization scheme. The bilayers were heated and thermalized in the *NVT* ensemble, and the structural, mechanical, and dynamical properties of thermalized bilayers were computed in the *NVE* ensemble.

*Analysis Using Convolutional Neural Netwroks*. Classification of crystalline phases (2H and 1T) and defects in strain-induced wrinkles, ridges, and Miura-Ori patterns in MoSe_2_ monolayers and bilayers is computationally expensive, especially for large systems consisting of millions of atoms. We address this challenge with a CNN, which is highly efficient and accurate in capturing the complexities of phase transformations and defect distributions in MoSe_2_ monolayers and bilayers.^48,49^

The CNN was trained by converting atomic coordinates to image-based data. The input data consist of 14 Å × 14 Å patches centered around every Mo atom. These patches are converted into 64 × 64 × 3 tensors using a 0.3 Å × 0.3 Å grid. Channels 1 and 3 of the input tensors correspond to the top and bottom Se layers, respectively, and channel 2 corresponds to the middle Mo layer. The atomic coordinates are transformed into a tensor using an exponential kernel
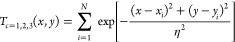
1where *c* refers to the input channel, (*x*, *y*) corresponds to the center of a grid, (*x*_*i*_, *y*_*i*_) are the coordinates of Mo and Se atoms, and *N* is the total number of atoms within a 14 Å × 14 Å patch around the central Mo atom. The width of the exponential kernel η is set to 0.2 Å.

The CNN architecture is shown in Figure S3. It consists of three convolutional layers with dimensions of 32 × 32 × 32, 64 × 16 × 16, and 64 × 8 × 8 and two fully connected layers with 4096 and 10 units. A softmax layer at the end outputs a number corresponding to 2H, 1T, and various defects. The stride size in each convolutional layer is 1, and the filter size is 3; we use a leakyReLU activation function (α = 0.2).

*Transfer Learning*. The CNN was trained on data from the simulation trajectory of crack propagation in a MoWSe_2_ alloy.^33,50,51^ The neural network could accurately identify not only 2H and 1T phases but also point and extended defects at 2H–1T interfaces in the alloy. The CNN trained with crack propagation data in MoWSe_2_ under tensile strain is used to analyze local structural changes induced by lateral compression in monolayer and bilayer MoSe_2_. The set of training data contains 40 000 simulation images of MoWSe_2_ under tensile strain and only 1000 images from the data set on MoSe_2_ monolayers. To validate CNN transfer learning, the trained neural network is tested on the remaining data sets of laterally compressed MoSe_2_ monolayers and bilayers. It correctly classifies atoms in 2H and 1T phases and chain-like defects that have been seen in STEM images of MoS_2_.^35^ The accuracy of the CNN on the test data sets is 97% (see Figure S3).

## Supporting Information Available

Force field parametrization and validation (Figure S1), convolutional neural network architecture and validation (Figure S2), structure and stress distribution in a uniaxially strained MoSe_2_ monolayer (Figure S3), wrinkle–ridge transformation in a MoSe_2_ bilayer (Figure S4), effect of strain on ridge formation in a MoSe_2_ bilayer (Figure S5), and energetics of ridges in MoSe_2_ bilayers (Figure S6) (PDF)
Biaxial compression of MoSe_2_ and emergence of Miura-Ori patterns (MP4)
Biaxial compression of MoSe_2_ and emergence of 2H and 1T phases (MP4)
