# Supplementary material for: Wrinkles, Ridges, Miura-Ori, and Moiré Patterns in MoSe2 Using Neural Networks
Source: J Phys Chem Lett. 2023 Feb 9;14(7):1732–9. doi: 10.1021/acs.jpclett.2c03539 (PMC9940294; doi:10.1021/acs.jpclett.2c03539)
Supplement: Supplementary file 1 — jz2c03539_si_001.pdf [file jz2c03539_si_001.pdf]

Wrinkles, Ridges, Miura-Ori, and Moiré Patterns in $MoSe_2$ Using Neural Networks

Anikeya Aditya[1], Ankit Mishra[1], Nitish Baradwaj[1], Ken-ichi Nomura[1], Aiichiro Nakano[1], Priya Vashishta[1], and Rajiv K. Kalia*[1]

[1]*Collaboratory for Advanced Computing and Simulations*
Department of Chemical Engineering and Materials Science, Department of Physics & Astronomy, and Department of Computer Science
University of Southern California, Los Angeles, CA 90089

I. Forcefield Parameterization and Validation

Stillinger-Weber Force Field (SWFF) [1] consists of pairwise interactions and 3-body angular terms. The total potential energy of a system of N atoms is expressed as,

$$V(r_1, r_2, \ldots, r_N) = \sum_{i<j} V_2(r_{ij}) + \sum_{i<j<k} V_3(r_{ij}, r_{ik}, \theta_{ijk}) \tag{1}$$

where $r_{ij} = |\boldsymbol{r}_i - \boldsymbol{r}_j|$, and θ_{ijk} is the angle between the ith atom located at $\boldsymbol{r}_i$ and its two nearest neighbors j and k at locations $\boldsymbol{r}_j$ and $\boldsymbol{r}_k$. The pairwise interaction, V_2, has the following form:

$$V_2(r_{ij}) = A\left(\frac{B}{r_{ij}^4} - 1\right) exp\left(\frac{\gamma}{r_{ij} - r_{cut}}\right) \tag{2}$$

where A, B and γ are optimizable parameters. The 3-body term,

$$V_3(r_{ij}, r_{jk}, \theta_{ijk}) = \lambda\, exp\left(\frac{\gamma_1}{r_{ij} - r_{cut1}} - \frac{\gamma_2}{r_{ik} - r_{cut2}}\right)(\cos(\theta) - \cos(\theta_0))^2 \tag{3}$$

also has three optimizable parameters (λ, γ_1 and γ_2) and interaction cutoff distances, r_{cut}, r_{cut1} and r_{cut2}. The angle, θ_0, is dictated by the crystal structure of $MoSe_2$ and is held fixed during optimization of parameters in the SWFF. The forcefield parameters are optimized against the crystal structure and phonon dispersion curves for $MoSe_2$, see Fig. S1. Ground-truth values of the lattice constant and phonon spectra are taken from density functional theory (DFT) calculations for the 2H and 1T' phases of the $MoSe_2$ monolayer.

The 2H phase of $MoSe_2$ monolayer is a honeycomb crystal structure and the hexagonal unit cell is composed of a layer of 6-fold coordinated Mo atoms sandwiched between two planes of 3-fold coordinated Se atoms in an ABA stacking. The crystal structure can be described by three two-body interactions (Mo-Mo, Se-Se and Mo-Se) and two three-body terms (Mo-Se-Mo and Se-Mo-Se). Excluding the interaction cutoff distances, the SWFF for $MoSe_2$ system has 13 design variables.

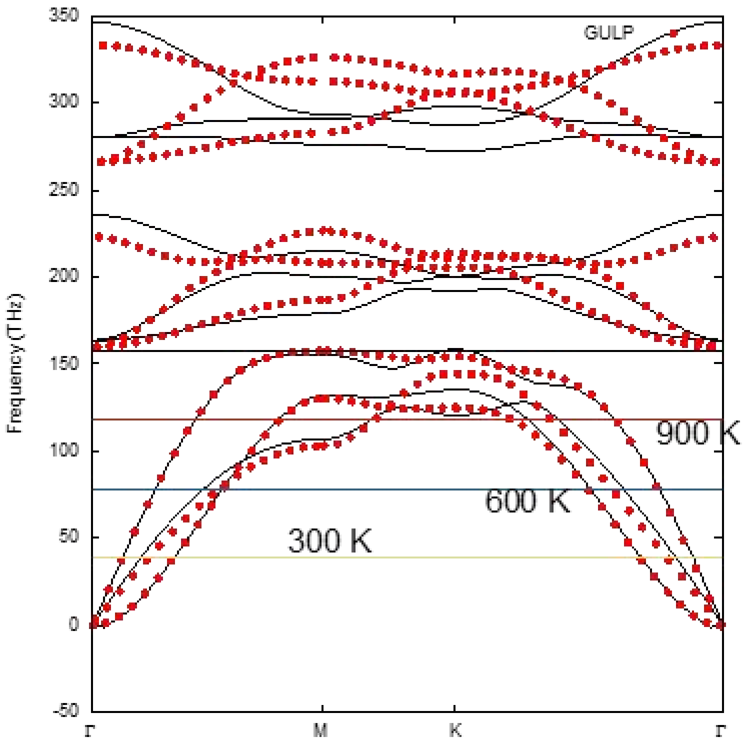

Figure S1: Phonon dispersion for an $MoSe_2$ monolayer along high symmetry directions in the Brillouin zone. Here the red dots are the values predicted by MD simulation using the optimized forcefield and the solid black line indicate the ground truth DFT values.

The forcefield parameters are optimized by genetic algorithms which depend upon population size, crossover, and mutation ratios, etc [2–5] . The forcefield was generated by the NSGA-III with a population size of 300 examples [6,7] . At every epoch, crossover was performed on 80% of the population using a simulated binary crossover operator. Mutation was simulated by choosing forcefield parameters from a uniform distribution between pre-specified lower- and upper-bounds for each decision variable. The optimized forcefield file MoSe2.sw is available per lammps specification.

II. Convolutional Neural Network (CNN)

CNN was used to classify 2H and 1T phases and defects [8] . The CNN architecture, shown in Fig. S2, consists of 3 CNN layers followed by a fully connected layer. The image generated by the CNN layer is fed to the fully connected layer to perform the classification task. Figures S2 (b) and (c) show that the network performs well on the training and test data. The loss functions are less than 0.1 after 25 epochs and the accuracy of the classified image is 0.99 for training and test data sets.

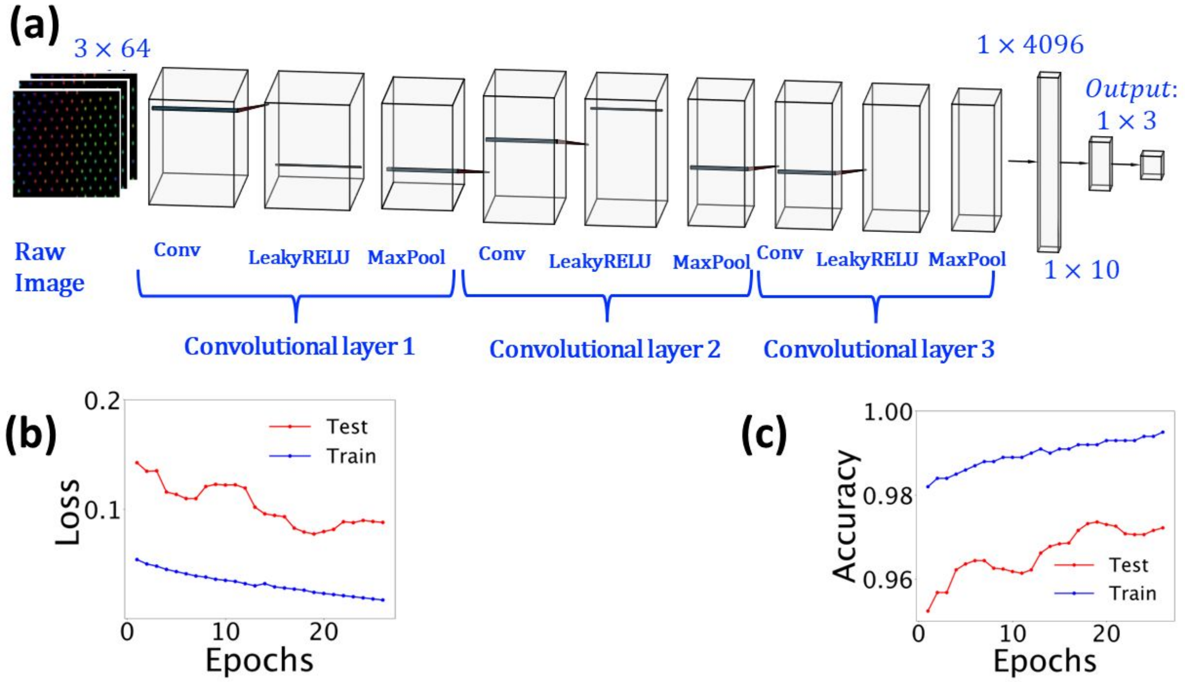

<u>Figure S2:</u> (a) CNN consists of 3 CONV layers, each containing Conv2D layers with the same padding and LeakyRELU and MaxPool layers. The CNN outputs a 3-dimensional vector corresponding to each image. (b) The loss evolution as a function of epoch shows that CNN performs reasonably well after 20 epochs and there is no overfitting on account of the test dataset. (c) The high accuracy on training and test datasets (99% and 97%) indicates high learning capability of the network.

III. Structure and Stress Distributions in a Uniaxially Strained $MoSe_2$ Monolayer

We have examined the behavior of a monolayer $MoSe_2$ at various values of compressive strain. Figure S3 shows the results at strains of 2, 6 and 10%. The top row in Fig. S3 shows the effect of strain on the structure of the monolayer. Dark blue color represents semiconducting 2H phase and light green color represents 1T metallic phase. The middle row shows how the stress distributions change with the applied strain on the monolayer. The 2H to 1T phase transformation occurs in regions of tensile strain. The last row in Fig. S3 shows the effect of strain on out-of-plane deformation in the $MoSe_2$ monolayer.

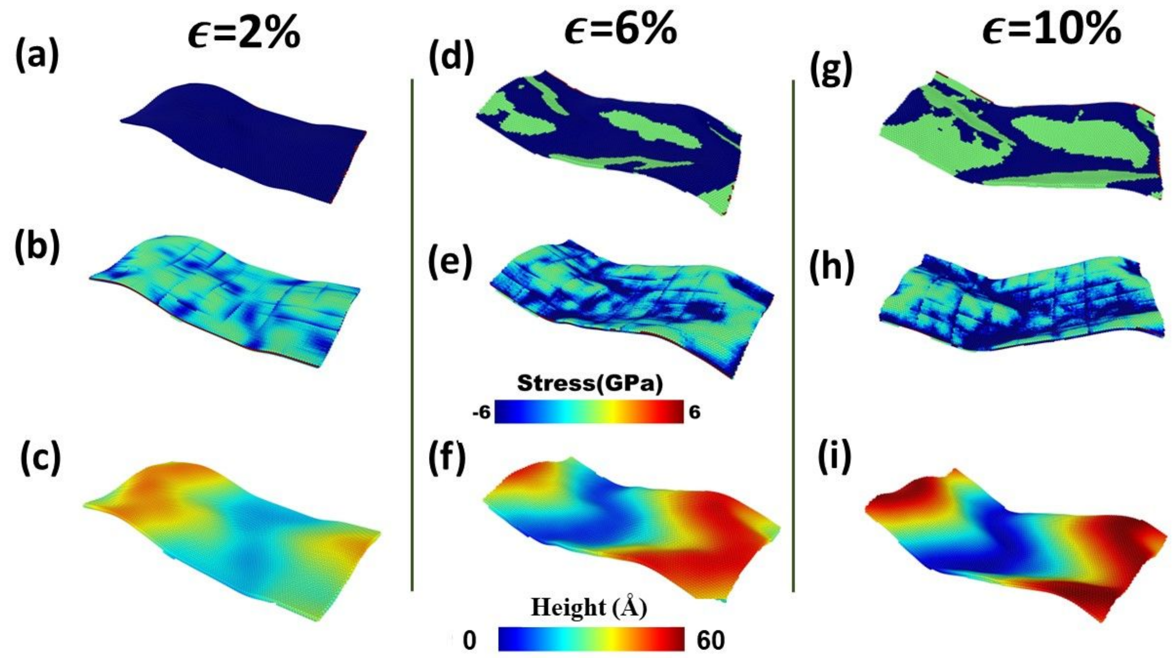

<u>Figure S3:</u> Shows the effect of strain on a monolayer of dimensions 400 × 200 Å under biaxial compression. The top row shows the distribution of 2H and 1T phases, the middle row shows the atomic stress distributions, and the bottom row shows the out-of-plane displacements of atoms under three different strains.

IV. Wrinkle-to-Ridge Transformation in an $MoSe_2$ bilayer

We study the effects of wrinkles and Miura-Ori folds on a pristine $MoSe_2$ monolayer. When we placed a pristine $MoSe_2$ sheet on a wrinkled $MoSe_2$ sheet and allowed the bilayer to relax under conjugate gradient scheme, the layers interact via van der Waals' forces and the wrinkles transform into a ridge. Figure S4 (a) shows the two layers at the start, and S4 (b) shows the wrinkle-to-ridge transformation. The wrinkled sheet is uniaxially compressed by 6%.

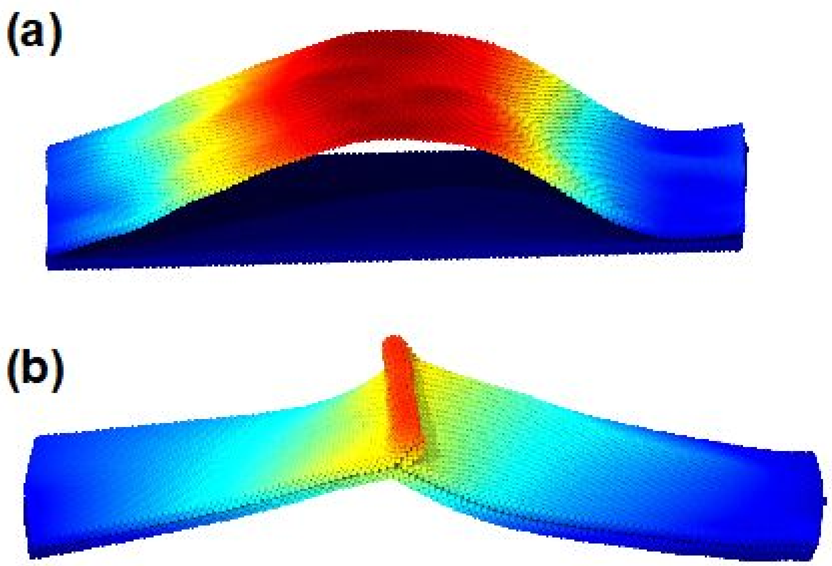

Figure S4: Shows the wrinkle-to-ridge transition in an $MoSe_2$ bilayer. The areal dimension of $MoSe_2$ sheets is 400 × 200 Å^2, and the applied uniaxial strain on the wrinkled sheet is 6% before a pristine $MoSe_2$ sheet is placed on top of the wrinkled sheet. Panel (a) shows the bilayer configuration before the van der Waals interaction induces the ridge formation shown in panel (b).

V. Effect of strain on ridge formation in an $MoSe_2$ bilayer

Figure S5 shows that compressive uniaxial strain has a significant effect on the size of the ridge. The ridge height doubles when the strain is increased from 4 to 10%. The monolayers are nearly flat away from the ridge, but they appear to form a nanotube near the ridge.

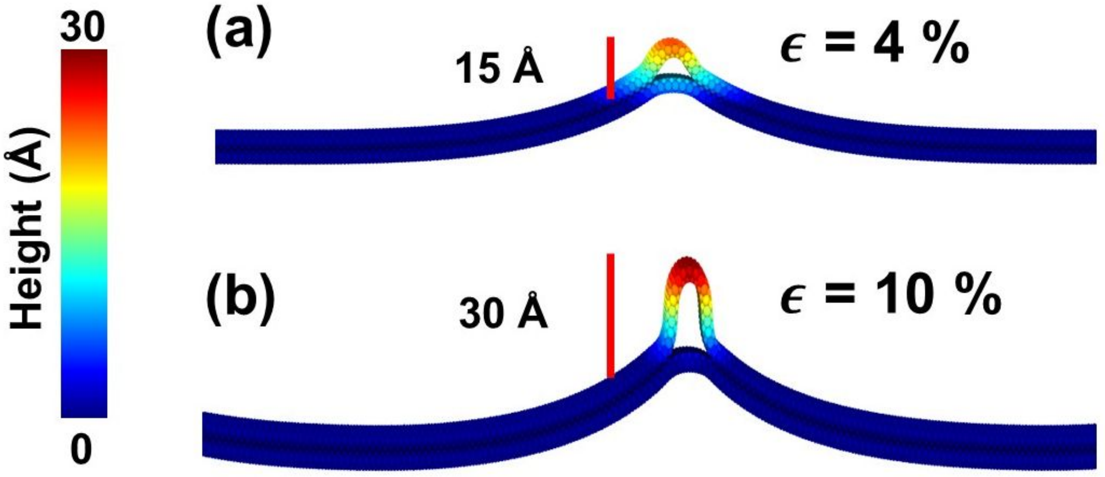

Figure S5: Shows the increase in ridge height with increasing strain from (a) 15 Å at 4% to (b) 30 Å at 10%. The system dimension is 400 × 200 Å^2.

VI. Energetics of Ridges in $MoSe_2$ bilayers

We also investigated how temperature affects the structure and energetics of the ridge. The first column of Fig S6 shows that the ridge height changes from 12 Å to 8 Å when the temperature increases from 100K to 200K. The second column shows the potential energy surface (PES) of the system at 100K and 200K. The PES is lower in the ridge than the flat region of the bilayer, and the drop in the potential energy of the ridge is higher at higher temperature.

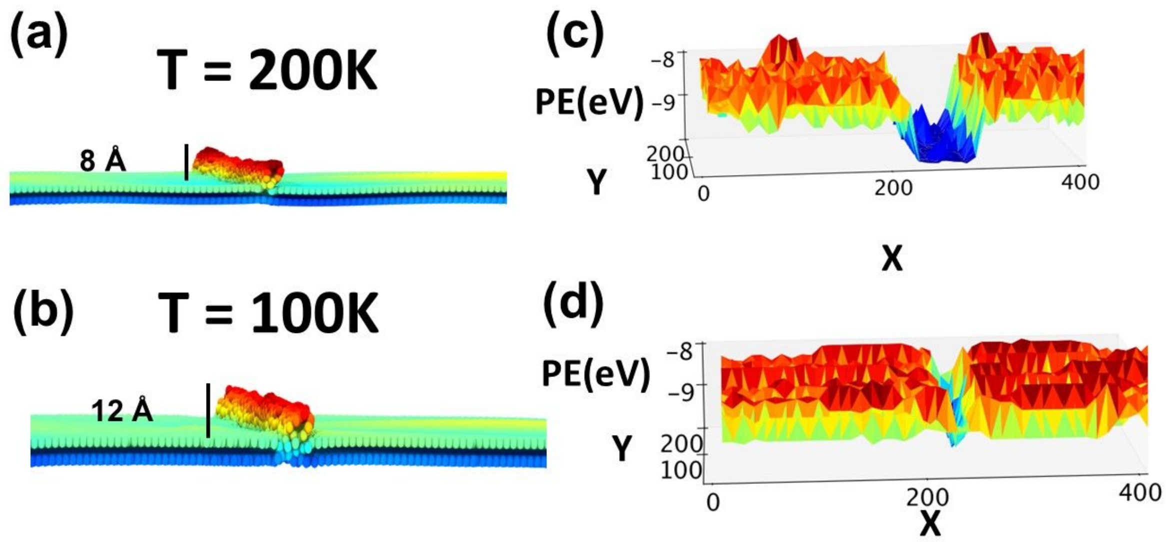

Figure S6: Effect of temperature on ridges in a uniaxially compressed $MoSe_2$ bilayer. The bilayer dimension is 400×200 Å^2, and the strain is 6%. Panel (a) and (b) show that heating lowers the ridge height. The potential energy surfaces at T = 100K and 200K are shown in panels (c) and (d), respectively. The potential energy valley in the ridge region becomes more distinct and wider with increasing temperature.

VII. Movies for MD Simulations

We have provided two movies for the MD simulations performed for the investigation. Movie 1 shows the out-of-plane deformation under biaxial compression. Dark blue color represents the valleys and red represent the peaks. Movie 2 shows the emergence of 1T phase from 2H phase along the slopes of the wrinkles as the sheet is compressed.

References

(1) Stillinger, F. H.; Weber, T. A. Computer Simulation of Local Order in Condensed Phases of Silicon. *Phys. Rev. B* **1985**, *31* (8), 5262–5271.

(2) Jaramillo-Botero, A.; Naserifar, S.; Goddard, W. A. General Multiobjective Force Field Optimization Framework, with Application to Reactive Force Fields for Silicon Carbide. *J Chem Theory Comput* **2014**, *10* (4), 1426–1439.
(3) Larentzos, J. P.; Rice, B. M.; Byrd, E. F. C.; Weingarten, N. S.; Lill, J. v. Parameterizing Complex Reactive Force Fields Using Multiple Objective Evolutionary Strategies (MOES). Part 1: ReaxFF Models for Cyclotrimethylene Trinitramine (RDX) and 1,1-Diamino-2,2-Dinitroethene (FOX-7). *J Chem Theory Comput* **2015**, *11* (2), 381–391.
(4) Ivanov, M. v; Talipov, M. R.; Timerghazin, Q. K. Genetic Algorithm Optimization of Point Charges in Force Field Development: Challenges and Insights. *J Phys Chem A* **2015**, *119* (8), 1422–1434.
(5) Mishra, A.; Hong, S.; Rajak, P.; Sheng, C.; Nomura, K.; Kalia, R. K.; Nakano, A.; Vashishta, P. Multiobjective Genetic Training and Uncertainty Quantification of Reactive Force Fields. *NPJ Comput Mater* **2018**, *4* (1), 42.
(6) Krishnamoorthy, A.; Mishra, A.; Grabar, N.; Baradwaj, N.; K. Kalia, R.; Nakano, A.; Vashishta, P. Evolutionary Multi-Objective Optimization and Pareto-Frontal Uncertainty Quantification of Interatomic Forcefields for Thermal Conductivity Simulations. *Comput Phys Commun* **2020**, *254*, 107337.
(7) Krishnamoorthy, A.; Mishra, A.; Kamal, D.; Hong, S.; Nomura, K.; Tiwari, S.; Nakano, A.; Kalia, R.; Ramprasad, R.; Vashishta, P. EZFF: Python Library for Multi-Objective Parameterization and Uncertainty Quantification of Interatomic Forcefields for Molecular Dynamics. *SoftwareX* **2021**, *13*, 100663.
(8) Krizhevsky, A.; Sutskever, I.; Hinton, G. E. ImageNet Classification with Deep Convolutional Neural Networks. In *Advances in Neural Information Processing Systems*; Pereira, F., Burges, C. J., Bottou, L., Weinberger, K. Q., Eds.; Curran Associates, Inc., 2012; Vol. 25.
